# Light-induced changes in the suprachiasmatic nucleus transcriptome regulated by the ERK/MAPK pathway

**DOI:** 10.1371/journal.pone.0249430

**Published:** 2021-06-30

**Authors:** Diego Alzate-Correa, Sydney Aten, Moray J. Campbell, Kari R. Hoyt, Karl Obrietan

**Affiliations:** 1 Division of Pharmaceutics and Pharmacology, Ohio State University, Columbus, OH, United States of America; 2 Department of Neuroscience, Ohio State University, Columbus, OH, United States of America; University of Lübeck, GERMANY

## Abstract

The mammalian master circadian pacemaker within the suprachiasmatic nucleus (SCN) maintains tight entrainment to the 24 hr light/dark cycle via a sophisticated clock-gated rhythm in the responsiveness of the oscillator to light. A central event in this light entrainment process appears to be the rapid induction of gene expression via the ERK/MAPK pathway. Here, we used RNA array-based profiling in combination with pharmacological disruption methods to examine the contribution of ERK/MAPK signaling to light-evoked gene expression. Transient photic stimulation during the circadian night, but not during the circadian day, triggered marked changes in gene expression, with early-night light predominately leading to increased gene expression and late-night light predominately leading to gene downregulation. Functional analysis revealed that light-regulated genes are involved in a diversity of physiological processes, including DNA transcription, RNA translation, mRNA processing, synaptic plasticity and circadian timing. The disruption of MAPK signaling led to a marked reduction in light-evoked gene regulation during the early night (32/52 genes) and late night (190/191 genes); further, MAPK signaling was found to gate gene expression across the circadian cycle. Together, these experiments reveal potentially important insights into the transcriptional-based mechanisms by which the ERK/MAPK pathway regulates circadian clock timing and light-evoked clock entrainment.

## Introduction

In mammals, the suprachiasmatic nucleus (SCN) of the hypothalamus functions as the master circadian oscillator. The rhythm generated by the SCN sets the phasing and regulates the synchrony of peripheral oscillator populations found in every organ system of the body [1, 2]. At a cellular level, circadian rhythms are generated by an auto-regulatory transcription/translation feedback loop that is built around a limited number of ‘clock genes’. Clock gene oscillations drive the rhythmic expression of thousands of clock-controlled genes, which, in turn, underlies the biochemical processes that give rise to rhythms in cellular physiology [3–6].

The phasing of the SCN oscillator is tightly regulated by the daily light cycle. This light-entrainment process is mediated by a monosynaptic input (via the retinohypothalamic tract) from intrinsically photosensitive retinal ganglion cells (ipRGCs) [7, 8]. In response to light, ipRGC nerve terminals release glutamate [9, 10] and pituitary adenylate cyclase-activating peptide (PACAP) from within the SCN [11], which in turn, actuates the entrainment process. One key feature of this entrainment system is that the resetting capacity of light is dependent on when the light stimulus is presented within the circadian cycle. For example, in dark-adapted rodents, transient exposure to light during the early subjective night causes a delay in the phase of the SCN rhythm, while light exposure during the late subjective night causes a phase advance of the rhythm; remarkably, the clock is not responsive to the same light stimulus if it is presented during the middle of the subjective day [12].

Within the SCN, photic stimulation is coupled to clock entrainment via a number of intracellular signaling pathways, including nitric oxide/protein kinase G (PKG) [13, 14]; calcium/calmodulin-dependent kinase II (CaMKII) [15, 16], and the p44/42 ERK/MAPK pathway [17, 18]. Of particular interest here is the p44/42 ERK/MAPK (or MAPK) signaling pathway. Light-evoked MAPK signaling tracks with the clock resetting effects of light: hence, the pathway is highly responsive to light input during the subjective night and is insensitive to light during the middle of the subjective day [17]. Additionally, disruption of MAPK signaling leads to an uncoupling of light from clock entrainment [18, 19]. Interestingly, signaling via the MAPK pathway shapes the response properties (e.g., the magnitude and direction of the phase shift) of the circadian clock to light [20]. Finally, the MAPK pathway is a potent regulator of inducible gene expression, and within the SCN, the pharmacological disruption of MAPK signaling leads to the suppression of a number of light-responsive immediate early genes [21].

Given the central role that light-evoked transcription likely plays in SCN clock entrainment, several groups have used RNA expression profiling approaches to examine the light-evoked transcriptome [22–24]; however, the contribution of MAPK signaling to this transcriptional response has not been examined. Here, we used microarray-based expression profiling to examine the role of MAPK signaling in the light-evoked transcriptional response. We focused on three circadian timepoints that define the unique, clock-time delimited response properties of the SCN to light: the subjective day, early subjective night, and late subjective night. The data presented here reveal a complex, context-specific role for MAPK signaling in the transcriptional profile of the SCN over the circadian cycle, and reveal that MAPK signaling is a key regulator of light inducible gene expression.

## Materials and methods

### Animal subjects

Unless specified otherwise, mice were grouped in home cages under a 12h light/12h dark (LD) cycle, with a temperature ranging between 18–23°C; 40–60% humidity, with water and food provided ad libitum. Equal numbers of female and male mice were used for all experimental groups. All animal care and experiments described here were performed in accordance with guidelines for animal experimentation at The Ohio State University and were approved by the Institutional Animal Care and Use Committee (IACUC).

### Cannulation, drug infusion and light treatment paradigms

Adult C57BL/6J wild type (WT) mice (8 to 14 weeks old) were initially anesthetized with ketamine hydrochloride (91 mg/kg) and xylazine (9 mg/kg), and then implanted with indwelling guide cannulae in the lateral ventricle using the surgical procedures outlined by Butcher et al. [18]. After the surgery, mice were individually housed under a 12 hour light/dark (L/D) cycle for a minimum of 2 weeks. Two days before infusion, mice were transferred to a 12h dark/12h dark (DD) cycle. After 2 days in DD, mice were infused (2 μL) with either drug vehicle, dimethyl sulfoxide (DMSO) or U0126 (10 mM in DMSO, Cell Signaling, #9903) 30 minutes prior to light treatment (or sham light treatment) at one of three time points. These time points were based on ‘zeitgeber times’ prior to transfer to DD, with a mid-day light pulse occurring at ZT 4, an early night light pulse occurring at ZT 15, and a late night light pulse occurring at ZT 22. For the infusion, mice were manually restrained under dim red light (<2 lux) and a 30-gauge injector needle was placed in the guide cannula and infusion was performed at a rate of 0.6 μL/min; the dummy cannula was returned to the guide cannula after infusion. Thirty minutes after infusion, mice were placed in a light-proof chamber and either exposed to light (100 lux for 10 minutes) or kept in the dark for the same amount of time (sham light groups). Finally, for immunohistochemical profiling of ERK activity, animals were euthanized 10 minutes after the end of the light/sham treatment; for RNA isolation, animals were euthanized 50 minutes after the end of the light/sham treatment.

### Immunohistochemistry

Mice were euthanized by cervical dislocation and decapitation under dim red light. Brain tissue was collected and transferred to ice-cold oxygenation media (NaCl 120 mM, KCl 3.5 mM, HEPES 10 mM, CaCl_2_ 0.5 mM, NaH_2_PO_4_ 1.2 mM, MgSO_4_ 2 mM, NaHCO_3_ 32.3 mM, glucose 10 mM), then 0.6 mm coronal tissue sections containing the SCN were obtained using a vibratome (Leica Biosystems Inc. VT1200. Buffalo Grove, IL). Sections were fixed in 4% paraformaldehyde for 12 hours at 4°C and then transferred to 30% sucrose solution in phosphate buffered saline (PBS) supplemented with sodium azide (2 mM) and sodium fluoride (3 mM) and then thin sections (40 μm) containing the SCN were obtained using a freezing microtome. Sections were then washed in PBS (3 times—5 minutes/per wash), permeabilized in 0.1% Triton X-100 in PBS (PBS-T: 30 minutes), incubated (15 minutes) in 0.3% H_2_O_2_ in PBST, blocked (2 hours) in 10% normal goat serum (NGS) in PBS-T, and incubated overnight at 4°C with the rabbit polyclonal primary antibody anti-Phospho-Erk1/2 MAPK (Erk1 Thr202/Tyr204; Erk2 Thr185/Tyr187) (1:500, Cell Signaling, #4370) prepared in 5% NGS in PBS-T. Next, sections were incubated (2 hours) at room temperature in 5% NGS in PBS-T with goat anti-rabbit biotinylated secondary antibody (1:500, Vector Laboratories) followed by incubation (1 hour) in avidin/biotin horseradish peroxidase complex (Vectastain Elite ABC labeling kit, Vector Laboratories). HRP labeling was detected using a diaminobenzidine-nickel-intensified substrate (Vector Laboratories). Finally, tissue was cleared with Xylene solution, and mounted on gelatin-coated slides with Permount media (Fisher Scientific, Houston, TX, USA).

### Cresyl violet staining

To visualize the cannulae placement, thin sections (40 μm) were mounted on gelatin-coated slides, dehydrated in a graded alcohol series, and then incubated in 0.1% cresyl violet solution for 15 minutes. Sections were then de-stained in 0.1% glacial acetic acid in 95% ethanol for 10 minutes, cleared in Xylene solution and immediately coverslipped with Permount.

### RNA isolation

Fifty minutes after the light/sham exposure mice were euthanized via cervical dislocation followed by decapitation under dim red light. SCN-containing brain tissue was processed into 0.6 mm coronal sections as described above. Sections were placed on microscope slides, flash frozen, and the SCN was dissected out under a stereomicroscope. SCN from two animals under the same treatment were pooled and stored at -80°C, and 3 independent pooled samples (biological triplicates) were prepared for each condition. RNA from each pooled sample was isolated by pestle disruption in 500 μL of Trizol (Invitrogen), which was followed by two rounds of chloroform extraction. The aqueous phase was mixed 1:1 with 70% ethanol and RNA was isolated using the RNAesy minikit columns (Qiagen), followed by wash steps per the manufacturer’s instructions. The RNA was eluted and resuspended in 20 μL of RNase Free water.

### Microarray hybridization and data analysis

For microarray hybridization, samples for each experimental group were prepared in triplicate, and each sample corresponds to pooled SCN tissue from two mice. Thus, for a total of 4 groups for three time points, we ran a total of 36 samples. RNA from each sample was quantified using a nanodrop 2000 (Thermo-Fisher) spectrophotometer followed by analysis of RNA integrity in an Agilent 2100 Bioanalyzer System (Agilent). Only RNA samples with a A260/A280 ratio >1.9; an A260/A230 ratio > 1.0 and a RIN score > 7 were used. Probes were prepared with 100 ng of total RNA as starting material using the GeneChip WT Plus Reagent Kit (Affymetrix, Santa Clara, CA); and hybridized to GeneChip Mouse Transcriptome Assay 1.0 following the manufacturer’s guidelines (Affymetrix, Santa Clara, CA). All analyses were undertaken using the R platform for statistical computing (version 3.1.0) and a range of library packages were implemented in Bioconductor. Global changes in mRNA in biological triplicate samples were analyzed by Oligo, and differential expression of genes (DEGs) were identified by a standard *limma* pipeline [25] using a generalized linear model and visualized by volcano plots (ggplot2) and heatmaps (pheatmap) [26]. Bracketing to the left of each heatmap denotes hierarchical gene expression clustering, which was performed using the Manhattan algorithm [27]. Genes with a P-value ≤ 0.1 and a fold change (FC) absolute value > 1.3 were considered statistically significant. All code is available upon request from the corresponding author. Finally, coding genes differentially expressed were clustered according to the functional annotation provided by the web-database DAVID (Database for Annotation, Visualization and Integrated Discovery) v6.8 [28]. Gene lists obtained for each comparison and timepoint were uploaded to the DAVID interface and compared against the *M*. *musculus* gene database. Enriched Functional Annotations and Functional Clusters were identified by using a threshold of less than 0.05 (Fischer Exact P-value). Finally, both Functional Annotations and Cluster were manually curated to eliminate redundant categories and a graphical representation of the Functional Clusters was generated using STRING functional protein association networks database [29]. Final figures were prepared with *Adobe Illustrator* Version 24.3 URL https://adobe.com/products/illustrator [30].

## Results

### Light-evoked MAPK pathway activation in the SCN

**Fig 1:**
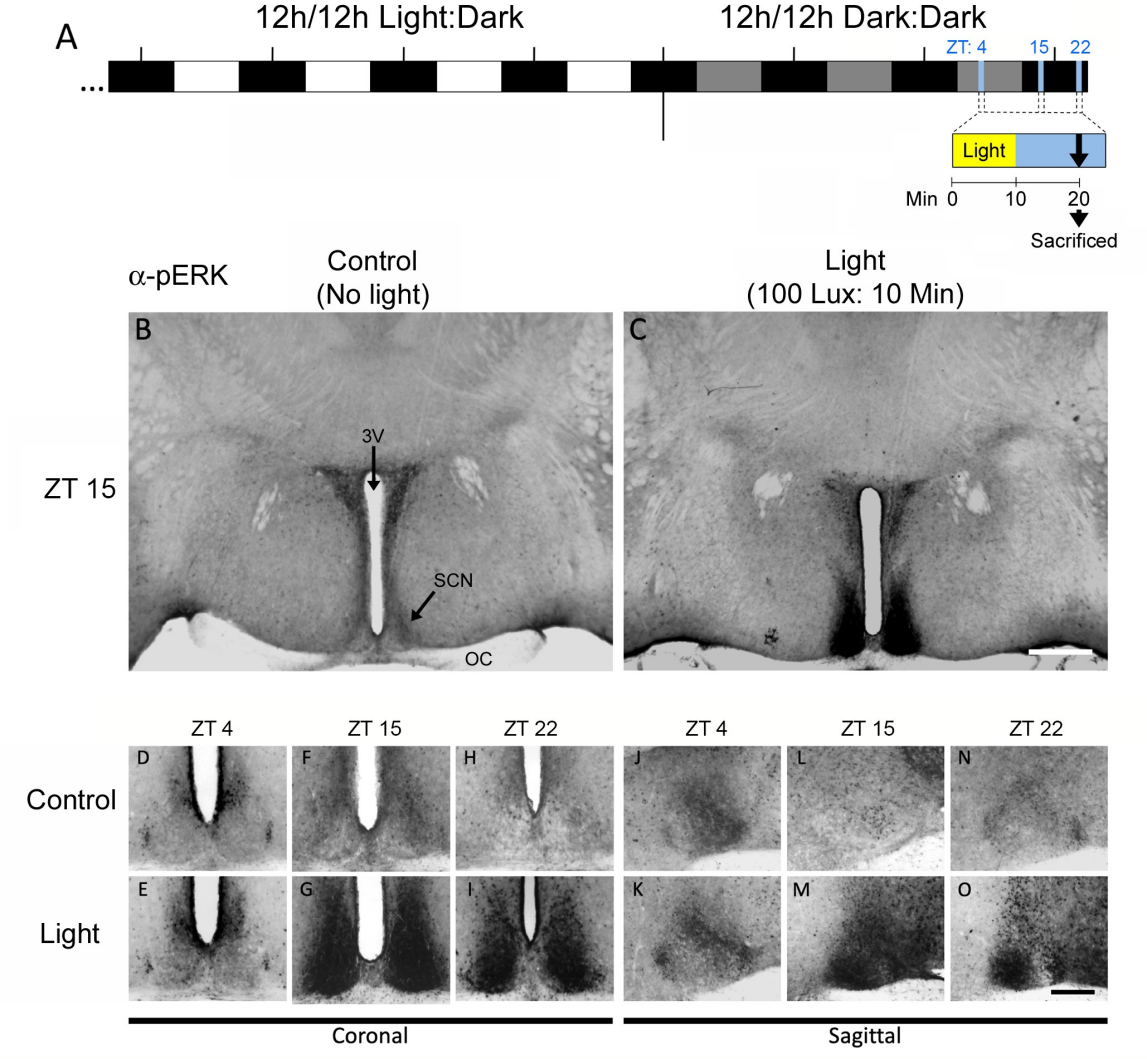
Light-evoked activation of ERK in the SCN. **A**. Schematic depiction of the experimental timeline used to 1) light-entrain and then transfer the mice to constant dark conditions, and 2) light pulse and sacrifice animals for immunohistochemical labeling. The timing of the light pulses was based on zeitgeber times prior to transfer to DD, and were designed to probe the light responsiveness of the SCN during the mid-day (ZT 4), early night (ZT 15) and late night (ZT 22). **B-C**. Representative SCN coronal sections from tissue collected at ZT 15 showing basal pERK levels (**B**) and pERK levels 20 minutes after the induction of a 10 minute, 100 lux, light pulse (**C**). Note the marked ERK activity specifically within the SCN. **D-I**. High magnification coronal images of pERK labeling within the central SCN at ZT 4 (**D**), ZT 15 (**F**) and ZT 22 (**H**), and pERK expression following photic stimulation at ZT 4 (**E**), ZT 15 (**G**) and ZT 22 (**I**). Note the light-evoked increase in pERK at ZT 15 (**G**) and ZT 22 (**I**), and the absence of marked induction at ZT 4 (**E**). **J-O**. SCN sagittal sections showing basal pERK levels at ZT 4 (**J**), ZT 15 (**L**) and ZT 22 (**N**), and light-induced levels at ZT 4 (**K**), ZT 15 (**M**) and ZT 22 (**O**). A similar pattern of pERK induction was observed at ZT 15 and ZT 22, whereas no increase is observed at ZT 4. Scale bar: 200 μm. OC: Optic chiasm; 3V: third ventricle.

As a starting point for our analysis of light-evoked, MAPK-dependent, gene expression, we performed a series of experiments to test both the light responsiveness of the MAPK pathway and the efficacy of the pharmacological disruption approaches. To this end, C57BL/6 mice were initially maintained using a standard, 12h light/12h dark, lighting condition and then transferred to constant darkness (DD: 12h dark/ 12h dark). DD removes the entraining effects of light and allows for the use of discrete light treatment paradigms during the middle of the subjective daytime. After two days in DD, mice were exposed to light (100 lux) for 10 minutes at one of three time points: mid-day, corresponding to zeitgeber time 4 (ZT 4), early night, corresponding to ZT15, or late night, corresponding to ZT 22; mice were then returned to darkness for 10 minutes and sacrificed (Fig 1A). Brain sections containing the SCN were collected, fixed and immunolabeled for the Thr202/Tyr204 phosphorylated form of ERK1 and the Thr185/Tyr187 phosphorylated form of ERK2 (collectively referred to as ‘phosphorylated ERK: pERK’) (Fig 1B and 1C). These dual phosphorylation events are a marker of ERK activation, and thus can be used as an index of MAPK pathway activity.

Consistent with prior work, light triggered a marked activation of ERK during both the early subjective night (ZT 15) and the late subjective night (ZT 22); low magnification images from the ZT 15 data point reveal that induction was largely localized to the SCN (Fig 1B and 1C), and higher magnification images in both the coronal and sagittal planes for both the ZT 15 (Fig 1F, 1G, 1L and 1M) and ZT 22 (Fig 1H, 1I, 1N and 1O) time points reveal that light-evoked MAPK activation occurred throughout the rostro-caudal extent of the SCN. In contrast, photic stimulation during the mid-day (ZT 4) did not trigger an increase in MAPK activity (Fig 1D, 1E, 1J and 1K). These results are in line with prior work showing that the MAPK pathway is responsive to photic stimulation during the circadian night, but insensitive to photic input during the subjective day [18].

### Pharmacological inhibition of MAPK signaling in the SCN

**Fig 2:**
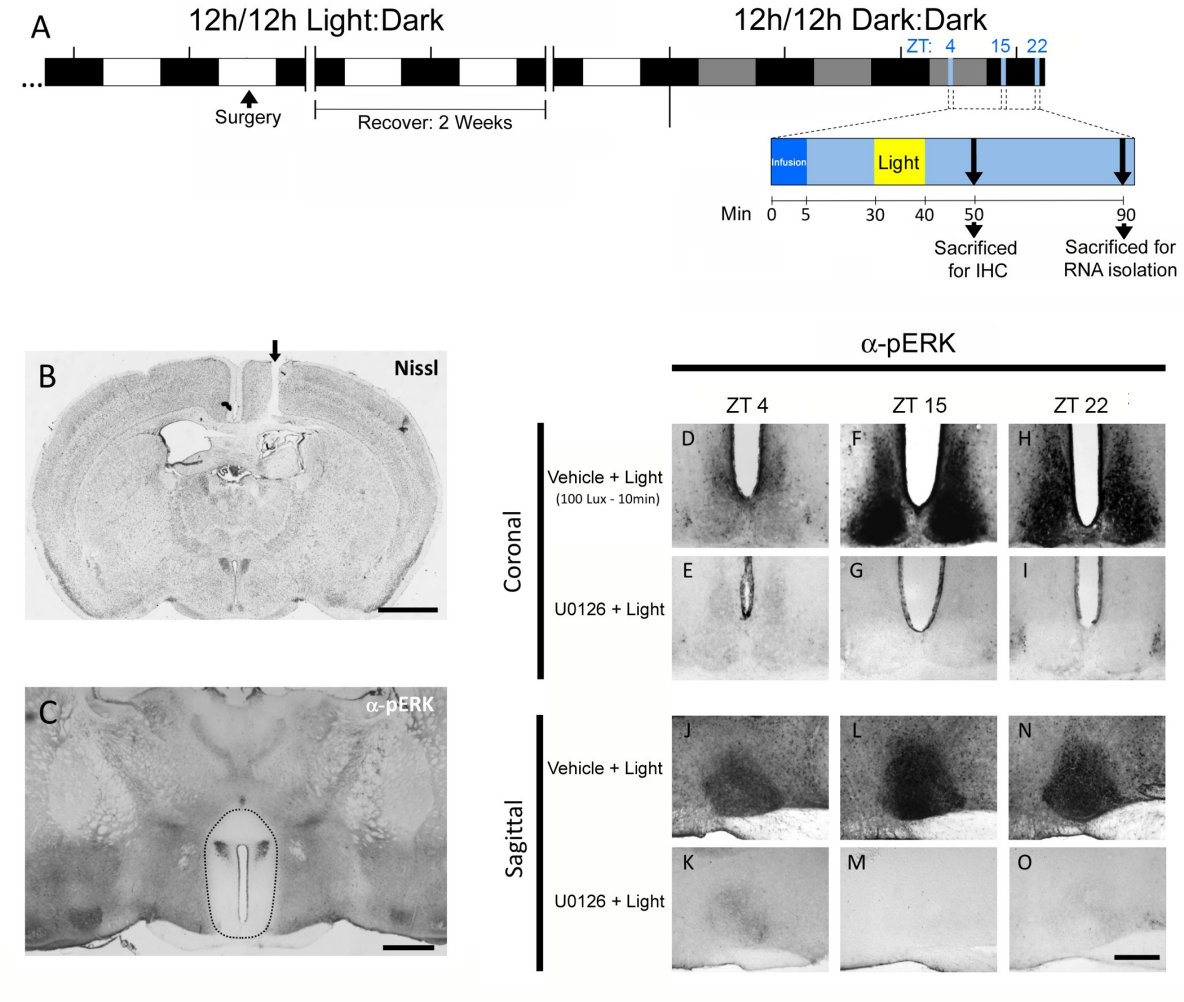
U0126-mediated suppression of MAPK signaling. **A**. Schematic depiction of the timeline used to 1) entrain and dark-adapt mice and 2) implant guide cannulae and 3) infuse, light pulse and sacrifice animals. **B**. Representative Nissl-stained coronal brain section showing the location of the cannula placement in the lateral ventricle. The arrow denotes the location of the scar left in the cortex by the guide cannula. **C**. Coronal brain section from an animal infused with U0126 that was immunolabeled for pERK; note the marked reduction in pERK expression within the periventricular region (denoted by the dashed line), which includes the SCN. (**D-I**) Representative coronal images showing pERK levels in the SCN after vehicle infusion and a light pulse (100 lux, 10 min) at each circadian time: ZT 4 (**D**), ZT 15 (**F**) and ZT 22 (**H**); and after infusion of U0126: ZT 4 (**E**), ZT 15 (**G**) and ZT 22 (**I**). (**J-O**) The effects of U0126 infusion are also presented using sagittal SCN sections for the three circadian time points: ZT 4 (**J:K**), ZT 15 (**L:M**) and ZT 22 (**N:O**). Scale bars: 1.5 mm for (**B**); 500 μm for (**C**); 200 μm for (**D-O**).

Next, we implemented an *in vivo* microinfusion approach (originally describe in Butcher et al. [18]) to deliver the MEK1/2 inhibitor U0126 to the SCN. Of note, MEK1/2 drives the phospho-activation of ERK1/2; as such, MEK1/2 inhibition should suppress ERK phosphorylation, and thus inhibit signaling via the MAPK pathway. Briefly, mice were implanted with guide cannulae targeting the lateral ventricle, allowed to recover for two weeks, dark-adapted (2 days) and then infused with either U0126 (10 mM) or drug vehicle (DMSO) thirty minutes prior to photic stimulation (100 lux, 10 minutes) or mock stimulation at ZT 4, ZT 15 or ZT 22. Mice were returned to darkness for 10 minutes and then processed for pERK expression as described above. The timeline of the experimental procedures is outlined in Fig 2A, and representative Nissl staining in Fig 2B is used to denote the location of cannula placement within the lateral ventricle.

The efficacy of U0126 to suppress MAPK activity is represented in Fig 2C, where a marked reduction in pERK labeling in hypothalamic regions surrounding the 3^rd^ ventricle was observed (the boundary of the reduced pERK labeling is denoted by the dashed ovoid). Importantly, the U0126 infusion paradigm led to a reduction in MAPK signaling under both the basal, no light, condition, and following light stimulation at each of the three circadian time points (Fig 2D–2O). With these paradigms in place, we turned to an examination of light-evoked, MAPK-dependent, gene regulation in the SCN.

### Light-dependent changes in the SCN transcriptome

To profile the light-evoked, clock-gated, transcriptional profile in the SCN, mice were infused with U0126 (or vehicle) and exposed to light (or mock light-treated) at the three noted time points (Fig 2A). After light exposure, animals were returned to darkness for 50 minutes (corresponding to 1 hour after the onset of light), and then sacrificed and total SCN RNA was extracted and prepared for microarray analysis.

**Fig 3:**
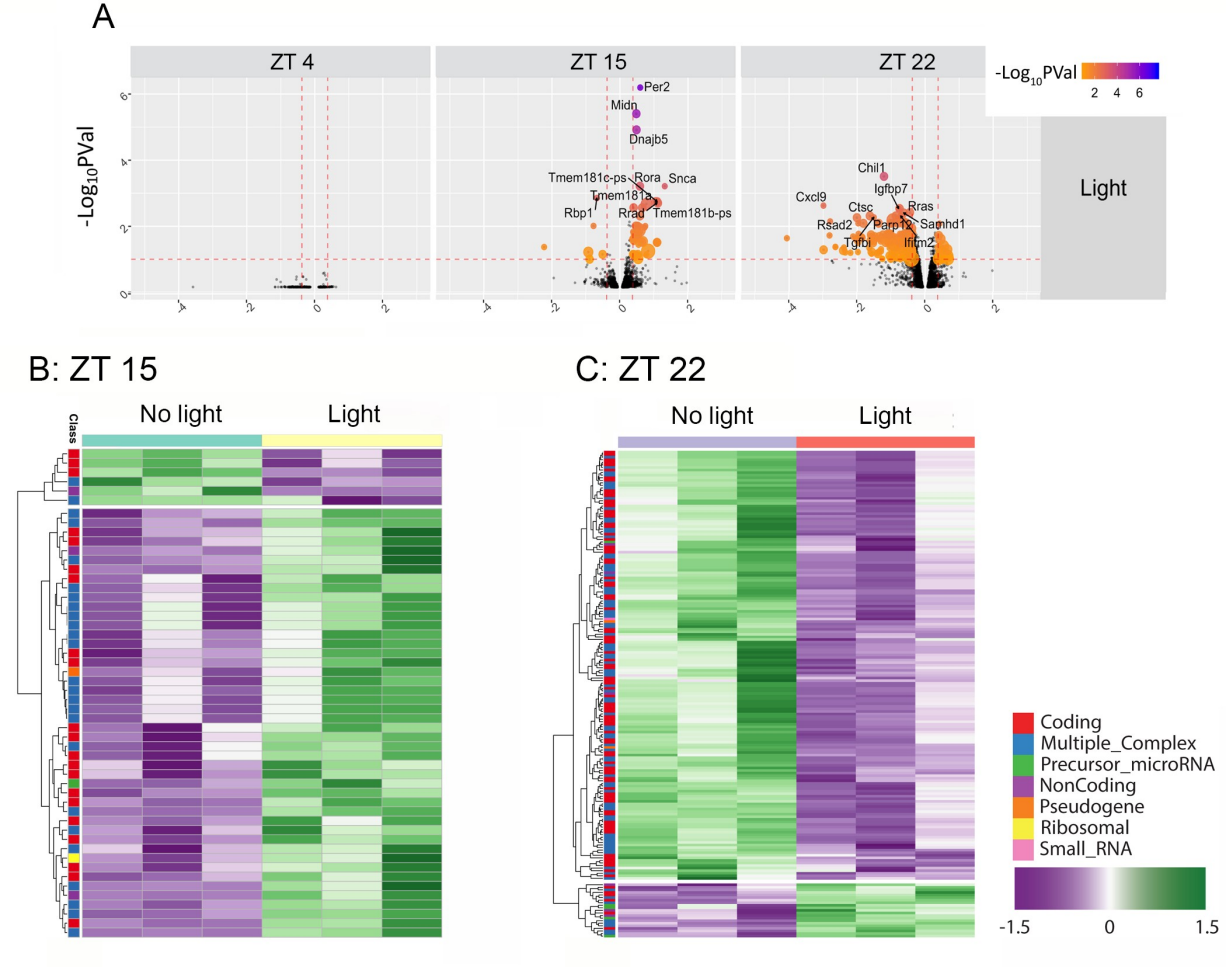
Bioinformatic analysis of light-evoked transcripts in the SCN. A. Volcano plot depicting light-induced changes in gene expression at three circadian time points: ZT 4 (left), ZT 15 (middle) and ZT 22 (right). Filtering criteria of absolute fold-change (FC) ≥ 1.3 and adjusted P-value (Pval) ≤ 0.1 (represented as -Log_10_PVal) are illustrated by dotted vertical and horizontal lines respectively. Included are the names of the top 10 transcripts showing statistically significant changes. With the noted filtering criteria we did not detect significant light-evoked changes in gene expression at ZT 4. **B-C**. Heat map clustering generated from the microarray data analysis (triplicate determinations for each condition) describing light-induced changes in gene expression at ZT 15 (**B**), and ZT 22 (**C**). The different transcript classes are color coded, and bracketing to the left denotes hierarchical gene expression clustering. Volcano plots and heatmaps were generated in *R* Version 3.1.0 URL https://www.R-project.org/ [53].

Initially, microarray hybridization data were used to identify transcriptomic changes in the SCN that were induced by light exposure at each circadian time point in our vehicle-infused animals (Fig 3A). Interestingly, at ZT 4, light did not trigger gene expression changes. Hence, no transcriptional changes (either increased or decreased expression) were detected 1 hour post-light treatment (Fig 3A). In contrast, photic stimulation at ZT 15 triggered a significant change (adjusted P-value > 0.1) in gene expression. Specifically, 46 genes (41 coding, 5 non-coding) were upregulated, and 6 genes (5 coding, 1 noncoding) were downregulated (Fig 3A and 3B; S1 Table). A parallel analysis for the late night, ZT 22, timepoint identified 191 differentially expressed genes (Fig 3A and 3C; S2 Table), with 21 up-regulated genes (14 coding, and 7 non-coding), and 170 downregulated genes (163 coding, 7 non-coding). Intersectional analysis of the light-evoked datasets identified only 1 gene (Rrad) that was regulated by light at both ZT 15 and ZT 22.

Finally, light-induction of core clock genes *period 1* and *period 2* is a well characterized phenomenon in the SCN. Here, we found that early night light led to an increase in *period 1* (1.29 fold; P-value = 0.051), and *period 2* expression (1.505-fold; P-value < 0.001), whereas during the late night, modest light-evoked induction of *period 1* and *period 2*, which did not reach statistical significance, was observed. Lastly, significant induction of the immediate early gene *c-fos*, which has also been found to be strongly induced by light, was not observed using our light stimulus strategy: potential reasons for this lack of responsiveness are outlined in the *Discussion* section.

### Gene ontology analysis

**Fig 4:**
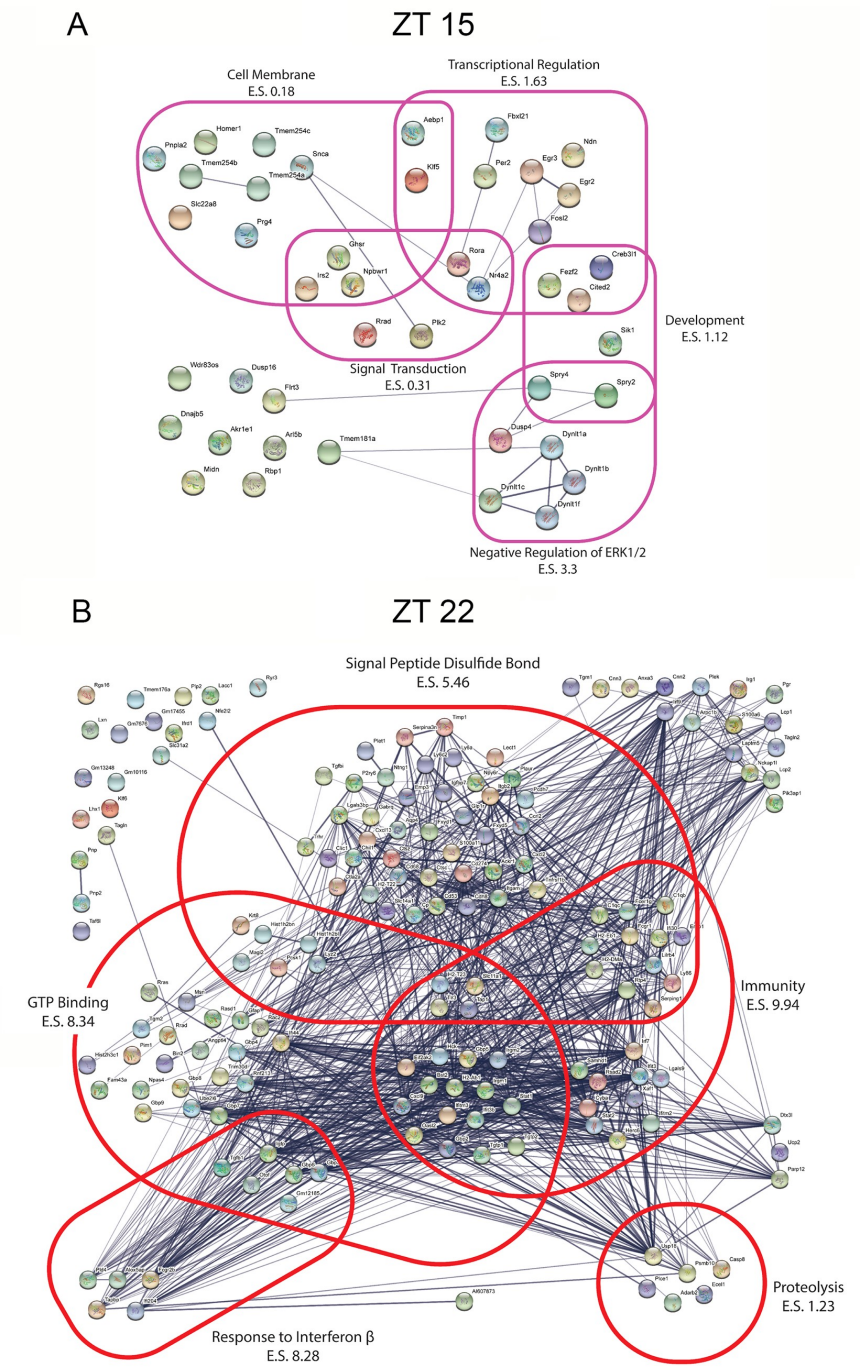
Functional enrichment clusters for transcripts induced by light. Ontological category clusters represented by the list of genes showing a statistically significant change after a light treatment at ZT 15 (**A**) and ZT 22 (**B**). Cluster and enrichment scores (E.S.) are generated using DAVID and are visualized using the STRING database visualization tool. Plots were generated in STRING database Version 11.0 URL https://string-db.org/ [51].

In order to explore the functional roles of the light-evoked gene sets, we performed an ontological enrichment analysis using the web-based database DAVID [31]. Analysis was not performed for the circadian day time point, given that light did not elicit a transcriptional response. In contrast, large and functionally diverse gene categories were affected by light at both ZT 15 and ZT 22. For ZT 15, 64 non-redundant functional categories were identified while at ZT 22 a total of 280 functional categories were identified. Visual representation of functional category interaction networks was prepared using the STRING functional protein association networks database [32]. With this approach,10 functional clusters were generated for the early night time point, including Negative Regulation of ERK1/2 Signaling; Transcriptional Regulation; Development; Cell Membrane, and Signal Transduction (Fig 4A). For the late night time point, 43 functional clusters were generated, including clusters related to Immunity, GTP Binding, Response to interferon β, Signal Peptide Disulfide Bond and Proteolysis (Fig 4B).

### MAPK suppression and the light-evoked transcriptome

Next, we examined the contribution of the MAPK pathway to light-regulated gene expression. To this end, we compared the noted light-evoked transcriptional profile to the light-evoked transcriptional profile following treatment with U0126. This intersectional analysis revealed that the disruption of MAPK signaling at the early night time point suppressed the expression of 32 of the 52 light-regulated transcripts (S3 Table). At the late-night time point the disruption of MAPK signaling suppressed the expression of 190 out of 191 of the light-regulated transcripts (S4 Table). Thus, MAPK signaling appears to play a critical role in coupling light to transcriptional activation in the SCN.

Interestingly, a comparative analysis of our light-treatment data sets (light pulse versus U0126 + light pulse) revealed that the suppression of MAPK signaling led to the expression of numerous genes that were not normally light responsive. Along these lines, at ZT 4, disruption of MAPK signaling triggered the light-evoked upregulation of 7 genes (6 coding and 1 non-coding: miRNA132/212) and the downregulation of 1 gene (ERDR1) (S5 Table). At ZT 15, disruption of MAPK signaling triggered the light-evoked upregulation of 45 genes (44 coding and 1 non-coding) and the downregulation of 18 genes (6 coding and 12 non-coding) (S6 Table), and at ZT 22, disruption of MAPK signaling led to the light-evoked upregulation of 7 genes (7 coding) (S7 Table). Together, these data reveal that MAPK signaling serves to dynamically regulate (i.e., both induce and suppress) gene expression as a function of light treatment and clock time.

### MAPK signaling and the clock-gated transcriptome

**Fig 5:**
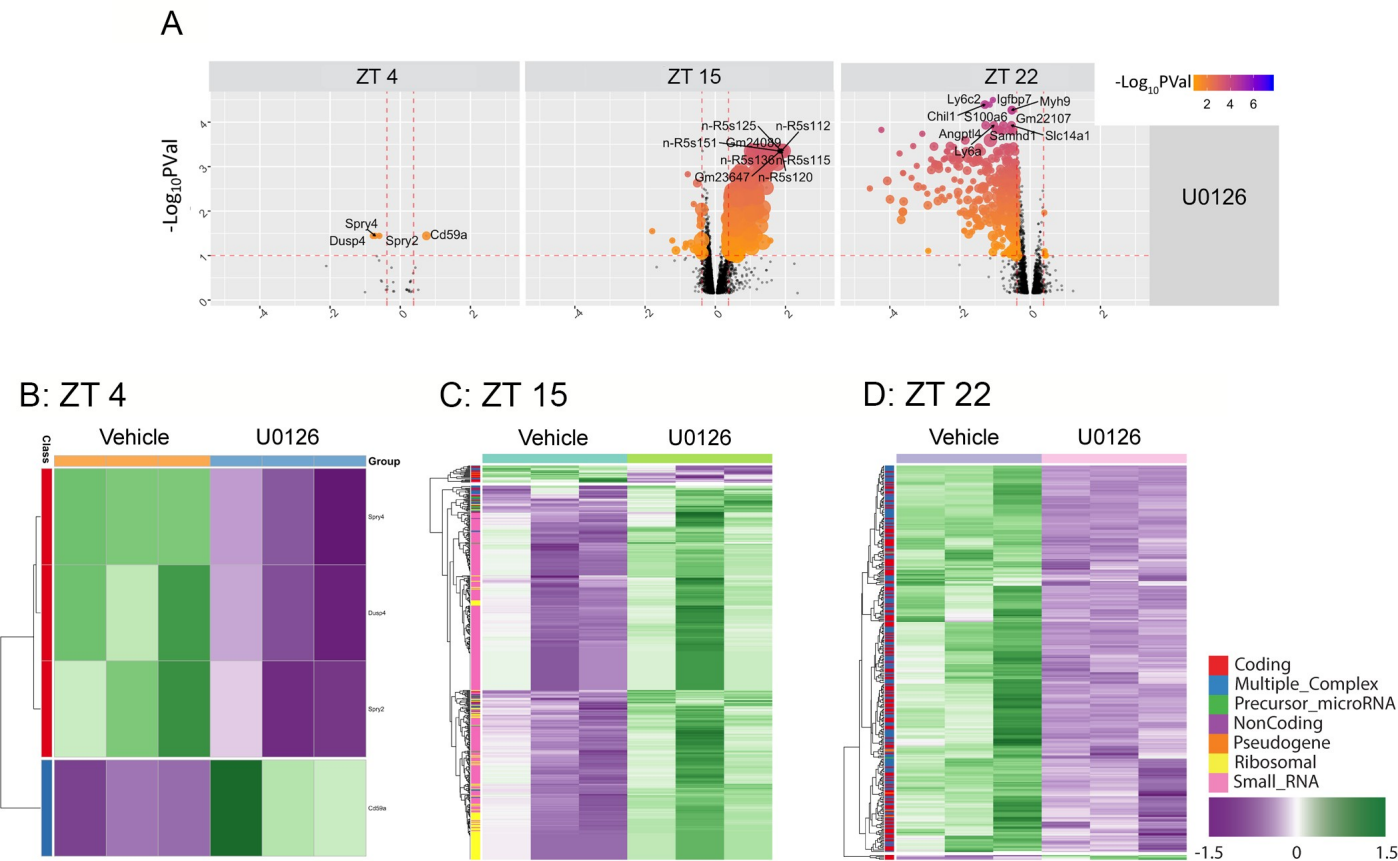
Bioinformatic-based analysis of MAPK-regulated transcripts in the SCN. A. Volcano plots depicting changes in gene expression induced by U0126 at mid-day (left), early night (middle) and late night (right). A fold-change (FC) ≥ 1.3 and adjusted P-value (Pval) ≤ 0.1 (represented as -Log_10_PVal) are illustrated by dotted vertical and horizontal lines respectively. Included are the names of the top 10 transcripts showing statistically significant changes. B-D. Heat map clustering (triplicate determinations for each condition) depicts U0126-mediated changes in gene expression at ZT 4 (B), ZT 15 (C) and ZT 22 (D). The different transcript classes are color coded and bracketing to the left denotes hierarchical gene expression clustering. Volcano plots and heatmaps were generated in *R* Version 3.1.0 URL https://www.R-project.org/ [53].

In another line of analysis, our control, no light, data sets were probed for the effects that MAPK inhibition had on the transcriptional profile at the three circadian time points. To this end, a comparison of the U0126- and the vehicle-treatment conditions revealed a marked effect that MAPK pathway disruption had on the SCN transcriptome. Along these lines, at ZT 4, MAPK suppression altered the expression of 4 coding genes. In specific, 1 gene (Cd59a) was upregulated, and 3 genes (Dusp4, Spry2, Spry4) were downregulated (Fig 5A and 5B; S8 Table). At ZT 15, MAPK suppression altered the expression of 506 genes. In specific, 484 genes (31 coding, 453 non-coding) genes were upregulated, and 22 genes were downregulated (18 coding, 4 non-coding) (Fig 5A and 5C; S9 Table). At ZT 22, MAPK suppression altered the expression of 335 genes, with 4 coding genes exhibiting increased expression, and 331 genes (318 coding, 13 noncoding) exhibiting downregulation (Fig 5A and 5D; S10 Table). Finally, intersectional analysis identified 1 gene (Spry4) that was altered at all three time points; and 6 genes that were altered at both early and late night time points (S11 Table).

**Fig 6:**
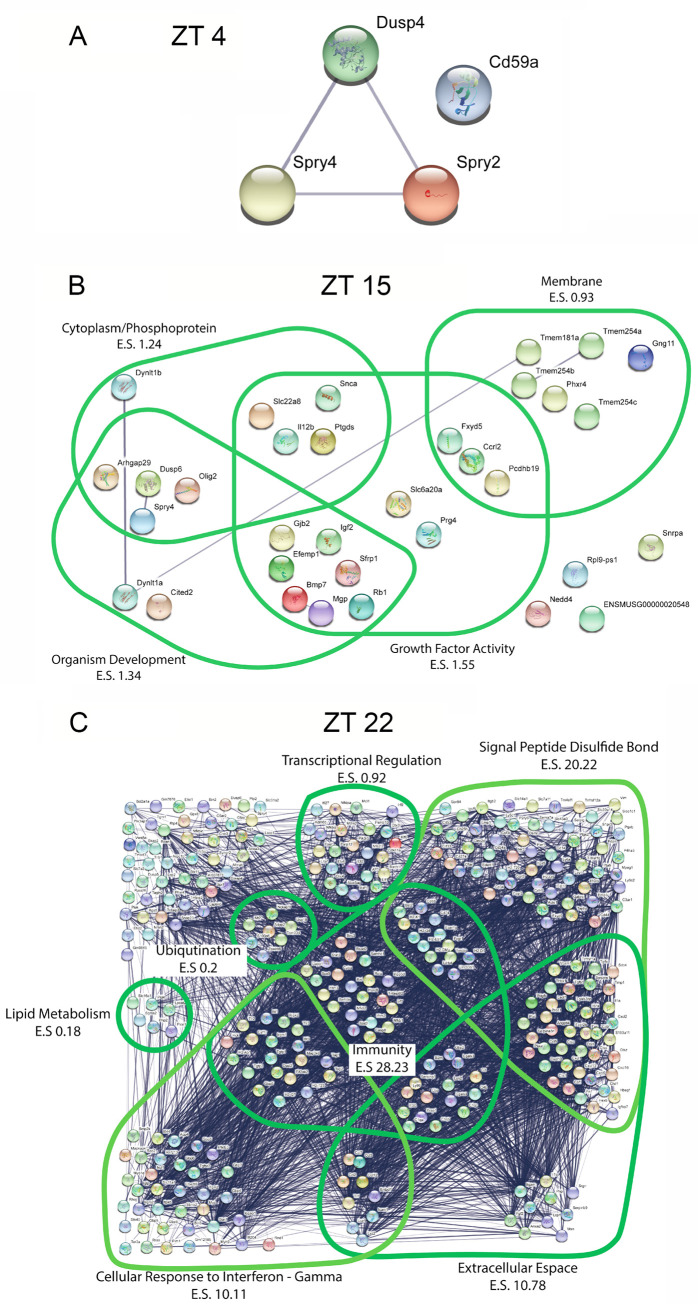
Functional enrichment clusters for transcripts regulated by MAPK signaling in the SCN. Ontological category clusters of the genes showing a statistically significant change after a light treatment at mid-day (**A**) early night (**B**) and late night (**C**). Cluster and enrichment scores (E.S.) are generated using DAVID and are visualized using the STRING database visualization tool. Plots were generated in STRING database Version 11.0 URL https://string-db.org/ [51].

DAVID ontological enrichment analysis was used to examine the effects of MAPK signaling on the basal transcriptional profile at the 3 noted circadian time points (Fig 6A–6C). Interestingly, distinct functional classes of genes were affected by MAPK inhibition at the early and late night time points. At the early night time point, 4 functional classes were identified, including growth factor signaling and membrane physiology (Fig 6B); at the late night time point, marked alterations in immunity genes were observed as were genes involved in transcriptional regulation, lipid metabolism and ubiquitination (Fig 6C). The relatively modest effects that MAPK inhibition has on gene expression during the subjective daytime, coupled with the largely distinct sets of genes affected during the early and the late night indicate that the circadian clock exerts potent, temporally delimited, regulation on MAPK transcriptional activity across the circadian cycle.

## Discussion

Here, we present the first report that examines the transcriptome-wide effects of MAPK signaling in the SCN. As a starting point for this study, we provide data that both supports and extends previous studies showing that, 1) photic stimulation leads to rapid ERK activation, 2) activation is restricted to the night time domain (corresponding to the time period when the circadian clock is sensitive to light) and 3) *in vivo* MEK inhibition potently suppresses MAPK activity and uncouples the clock from light. With the use of a comprehensive mouse gene expression assay that covers both coding and noncoding transcripts, we further this line of work by showing that the MAPK pathway contributes to both the clock-gated and light-evoked transcriptional program within the SCN.

### Light-evoked, clock-gated, gene expression

As a starting point for the gene profiling studies, we reported on the light-evoked transcriptional response at three circadian time points that are representative of the unique, time-domain-specific response properties of the SCN: the early circadian night (phase delaying), the late circadian night (phase advancing) and the circadian day (light refractory). The key observations that came from these studies are that 1) gene induction does not occur during the middle of the circadian day, and 2) light triggers largely distinct gene expression patterns during the early and late subjective night.

Our finding that light does not affect gene expression during the subjective day is largely consistent with work over the past ~ 30 years showing that light-evoked gene expression is limited to the night time domain. Along these lines, immediate early genes (IEGs), such as c-*fos*, *junB*, *egr-1* and the core clock genes *period1 and period2* exhibit rapid expression following light exposure during the night time domain, but not during the subjective day [33–39]. However, it is notable that a recent transcriptome profiling study by Hatori et al. (2014) reported a modest transcriptional response at 1 hour after CT 6 light exposure (9 upregulated and 2 downregulated/suppressed transcripts, with a greater number of genes observed at 2 hours post-light treatment) [22]. The difference between our profiling data and the work of Hatori et al. [22] could be ascribed to a wide array of differences in experimental conditions and data collection and analysis methods.

Some of the well-characterized light-evoked IEGs (e.g., c-*fos*, *junB*, *egr-1*) were not detected in our array data set. One likely explanation for this is the short half-life of IEG transcripts [40]. As such, the 1 hour-post light treatment time point used to collect the tissue likely did not provide the temporal resolution required to detect rapidly induced, and transiently expressed, IEG transcripts. Further, though the 1-hr time point did detect a large number of light evoked transcripts, the relatively modest induction of some transcripts (such as *period 1*) may be a function of the time course of profiling or our SCN tissue isolation method, which, for genes that are induced in only a fraction of SCN cells, would lead to a markedly attenuated signal. Additional approaches, including laser capture microdissection or single cell RNA sequencing would certainly help determine the response profiles of distinct neuronal and non-neuronal cell populations. With that said, our profiling identified a large number of transcripts that have been detected in other studies, including *Per2*, *Rora*, *Rrad*, *Egr2*, *Egr3*, *Sik1*, *Nr4a2*, *Fosl1*, *Spry4*, *Arl5b*, *Klf5*, *Midn*, *Plk2*, *Dusp4*, *Cdh8*, *Trhr*, *Ntng1* and *miR212/132* [22, 23]. Finally, in addition to the noted potential explanations, the lack of a significant induction of *period* genes during the late night could also be related to the limited sensitivity of the clock to light. In specific, our late-night ZT 22 light treatment was based on the LD cycle prior to transfer to DD; however given that the tau of C57BL/6J mice is slightly shorter than 24 hrs (23.77: [41]) and that mice were transferred to DD for 2 days, the circadian time for the light pulses may have been close to 22.5 (of note, circadian timing was not independently confirmed for the animals used in this study). At this point in the circadian cycle (circadian time 22.5), prior photic phase-response profiling studies have shown that the SCN clock in C57Bl6J mice has limited sensitivity to light [42, 43]. *Period* genes are state variables of the circadian oscillator, and as such, our data showing limited light sensitivity of *period* transcripts at this late night time point would appear to be consistent with the limited photic sensitivity of the clock as it transitions from the circadian night to the circadian day.

Comparing the early and late night time points revealed a marked divergence in the number and functional classes of genes that were regulated by light. Further, functional analysis of the differentially regulated genes at these two circadian times revealed distinct enriched ontological clusters. To this point, only one gene was regulated by light at both time points and there were no common ontological clusters. Further, it was interesting to find that the profiles of light-evoked genes biased toward increased expression during the early night and reduced expression during the late night. What might it mean that such large and functionally distinct sets of genes are regulated in response to early and late night light? Could the induction of such a diversity of genes underlie the magnitude of the phase shift, or might the differences in relative expression of induced and repressed genes at these time points contribute to the differential response properties of the SCN to early night and late night light (i.e., phase delaying and phase advancing, respectively)? Although there have been several studies that have examined the underlying molecular mechanisms (e.g., gene expression patterns and signal transduction networks) that drive the differential response properties of the SCN to early night and late night light [20, 44–46], at this point, there is no simple or direct answer to this question. Given the diversity of inducible genes and distinct transcriptional responses to light during the early and late night reported here, it is tempting to speculate that the precise light-evoked, phase-specific, response properties of the clock are dependent on the concerted effects of a wide array of gene products, rather than the effects of a single gene/gene family (e.g., *period genes*), or a small subset of genes.

### MAPK-dependent gene expression

Although numerous studies have shown that the MAPK-mediated signaling regulates both basal and inducible neuronal gene expression [47, 48], we were surprised by the degree to which light-evoked gene expression in the SCN was dependent on MAPK signaling. Along these lines, MAPK pathway disruption repressed light-regulation of over 60% of the early night genes and nearly all light-responsive genes during the late night. To a certain extent, these potent effects are consistent with prior studies showing U0126 infusion suppressed the expression of immediate early genes (i.e., c-Fos, JunB, EGR-1) during the early night in the SCN [21]. When these data are considered within the context of work showing that light-evoked MAPK signaling is temporally correlated with the time periods when the clock is sensitive to light (i.e., the night time domain), and work showing that MAPK signaling is essential for light-evoked clock entrainment, these data indicate that MAPK-dependent gene transcription is a key event in the entrainment process.

Here, it is also worth noting that, although MAPK signaling is necessary for clock entrainment, it is unlikely to be sufficient, or it may not even be uniquely positioned as an essential regulator of light-evoked clock entrainment. Along these lines, a good number of additional kinase pathways have been shown to be light responsive, including nitric oxide/protein kinase G (PKG) [13, 14]; calcium-calmodulin kinases (CaMKs) [15, 16]. These pathways may function independently of the MAPK pathway to regulate inducible gene expression, and/or they could function in coordination with the MAPK pathway as is the case for CaMKII [18] to affect clock entrainment.

### Circadian regulation of MAPK signaling

In addition to its robust responsiveness to light, the activation state of the MAPK cascade is also modulated over the circadian cycle [18]. Along these lines, peak levels of ERK activation are detected during the middle of the subjective day (when MAPK signaling is not activated by light) and low levels of ERK activity are detected during the night time domain. Although the experiments described here were designed to examine the role of the MAPK pathway in light-evoked gene expression, our control data sets, where mice were infused with U0126 in the absence of light, provided an interesting set of insights into the roles that MAPK signaling plays in circadian-gated gene expression. Notably, the suppression of MAPK signaling altered the expression of a large number of genes during the night time domain (506 during the early nighttime and 331 during the late night). The fact that intersectional analysis only identified a limited number of genes that are regulated by MAPK signaling during both the early and late night raises the prospect that MAPK signaling may play a critical role in gating key functional properties of the SCN oscillator as it progresses across the circadian night. Consistent with this idea, suppression of MAPK signaling has been shown to disrupt the timing properties of the SCN [49, 50]. Potential MAPK-regulated genes that could affect clock timing include a wide variety of channels, transcription factors and genes that play a role in cellular plasticity. Finally, the disruption of MAPK signaling had minimal effects on gene expression during the daytime, even though marked ERK activation is detected through the SCN during the circadian day [17]. Clearly, the underlying mechanisms that regulate time-of-day transcriptional regulation by the MAPK pathway merits further investigation.

### MAPK-mediated suppression of gene expression

Interestingly, in addition to our data showing that MAPK signaling is a principal route by which inducible gene up-regulation occurs, our data also revealed that MAPK signaling functions as a potent repressor of gene expression. MAPK-mediated suppression of gene expression has been most thoroughly explored within the realm of cell cycle control mechanisms [51]. For example, chronic ERK activation has been shown to lead to the downregulation of antiproliferative genes [52]. At a mechanistic level, several studies have identified potential routes by which this could occur. For example, Stat3-dependent transcription has been shown to be repressed by ERK activation [53]. Likewise, ERK was recently found to phosphorylate the cofactor MCRIP1, which in turn leads to CtBP-mediated gene silencing [54]. Hence, within the SCN, there appears to be a complex interplay of mechanisms by which the MAPK pathway modulates (both positively and negatively) the light-evoked transcriptional profile in the SCN.

Finally, beyond transcriptional regulation, the MAPK pathway has complex and diverse post-transcriptional effects that likely also contribute to its effects on clock timing and entrainment. For example, MAPK signaling has been shown to modulate mRNA translation via the mTOR pathway in the SCN [55, 56]. Further, via its post-translational effects within the cytoplasm, MAPK signaling regulates cell excitability and cell morphology [57–59]: two processes that could also contribute to the resetting effects of light. However, at its core, light-evoked clock resetting appears to require transcriptional activation. As such, the data presented here provide a novel exploration of a key signaling pathway that drives transcriptional activity and the light-evoked resetting of the molecular clock.

## Supporting information

S1 Table ZT 15: Light-regulated genes. (XLSX)

S2 Table ZT 22: Light-regulated genes. (XLSX)

S3 Table ZT 15: Light-regulated genes that were suppressed by U0126. (XLSX)

S4 Table ZT 12: Light-regulated genes that were suppressed by U0126. (XLSX)

S5 Table ZT 4: List of genes that were regulated by light following MAPK suppression. (XLSX)

S6 Table ZT 15: List of genes that were regulated by light following MAPK suppression. (XLSX)

S7 Table ZT 22: List of genes that were regulated by light following MAPK suppression. (XLSX)

S8 Table ZT 4: List of genes that were regulated following MAPK suppression. (XLSX)

S9 Table ZT 15: List of genes that were regulated following MAPK suppression. (XLSX)

S10 Table ZT 22: List of genes that were regulated following MAPK suppression. (XLSX)

S11 Table List of genes that were regulated following MAPK suppression at both ZT 15 and ZT 22. (XLSX)
